# Spatio-temporal expression of E-cadherin mediates trophoblast lineage transitions during human placentation

**DOI:** 10.1007/s11033-026-12837-0

**Published:** 2026-10-08

**Authors:** Enoch Appiah Adu-Gyamfi, Ethan Backes

**Affiliations:** Department of Biology, University of Wisconsin-Stout, 410 10th Avenue E, Menomonie, Dunn County, Menomonie, 54751 WI USA

**Keywords:** E-cadherin, Trophoblast, Placenta, Pregnancy, Preeclampsia

## Abstract

Human placentation relies on the precise differentiation of embryo-derived trophoblasts into specialized lineages, a process strictly regulated by the spatio-temporal expression of key genes. Herein, we report that the expression of the transmembrane glycoprotein, known as E-cadherin, fluctuates dynamically across distinct trophoblast subpopulations rather than acting as a static structural tether. Along the villous pathway, E-cadherin expression initially increases to facilitate the aggregation of cytotrophoblast cells (CTs) and is subsequently downregulated via transcriptional repression, enzymatic cleavage and physical compressive forces for the CTs to fuse into the syncytiotrophoblast lineage. Conversely, along the extravillous pathway, its transient downregulation characterizes an epithelial-to-mesenchymal transition that permits trophoblast invasion into the decidua, followed by its upregulation in the endovascular trophoblasts to potentially facilitate vascular adhesion and spiral artery remodeling. Dysregulation of this expression pattern of E-cadherin is evident in the placenta of pregnancies complicated by preeclampsia or fetal growth restriction. To advance the field, future research must transition from descriptive profiling to functional mechanisms by defining the precise intracellular degradation pathways of E-cadherin in trophoblast lineages and then clarify whether the upstream regulators operate in linear or parallel networks. Utilizing advanced biophysical tools and spatial transcriptomics will help clarify how tissue stiffness, fluid shear stress, and local immune cells mechanically and immunologically trigger this E-cadherin switch via mechanosensitive channels. Translational studies should explore the feasibility of using circulating soluble E-cadherin fragments in maternal blood as an early biomarker of defective placentation.

## Introduction

Placentation refers to the formation, growth and structural arrangement of the temporary but vital organ known as the placenta. Once formed, the placenta supports fetal development by facilitating gas and nutrient exchange, eliminating metabolic waste products, producing pregnancy-sustaining hormones, and establishing feto-maternal immune tolerance. Therefore, placentation is a crucial process; impairments of which have been implicated in gestational pathologies such as miscarriage, preeclampsia and fetal growth restriction. The process of placentation involves the differentiation of embryo-derived cells called trophoblasts into specific lineages that perform specialized functions. Such lineage specifications are controlled by the time-course expression of key genes.

Epithelial cadherin (E-cadherin) is classically known for maintaining epithelial cohesiveness, while its dynamic expression during human placentation has been reported as a molecular component of trophoblast lineage transitions. As trophoblast progenitors differentiate into highly specialized lineages, E-cadherin expression changes to facilitate these processes. Despite the reported involvement of E-cadherin in placental morpho-physiology and pathology, the current literature remains fragmented across studies and lacks a unified framework to bridge emerging insights from mechanobiology and spatial transcriptomics. Hence, this review summarizes current literature on E-cadherin dynamics in trophoblasts, highlights existing knowledge gaps, and proposes future research directions to provide a comprehensive framework for its role in human placentation.

## Overview of human placentation

**Fig. 1:**
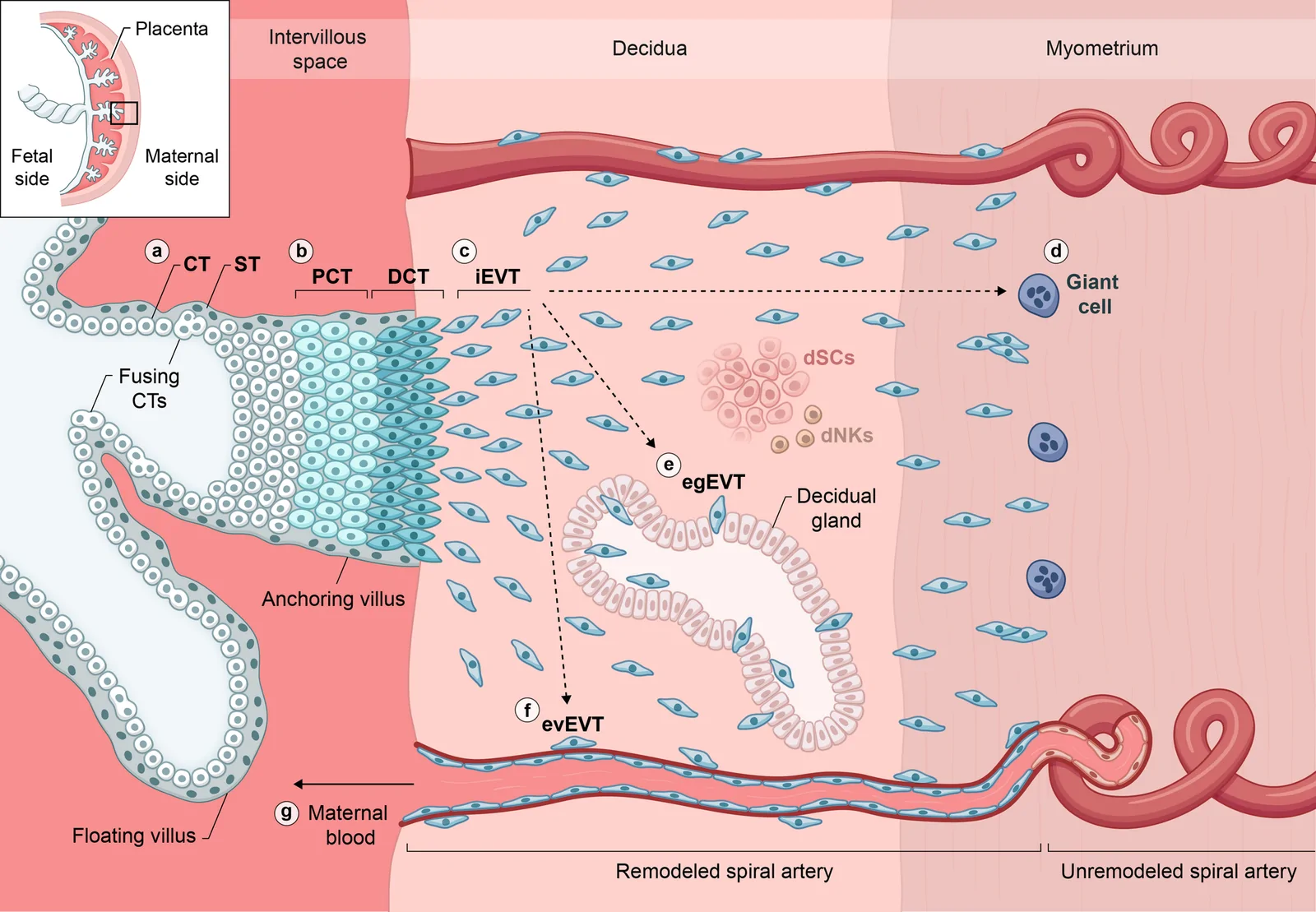
Schematic overview of human placentation. After implantation, the trophectoderm of the embryo gives rise to CTs, which differentiate into two major trophoblast subtypes: the STs via a fusion process called syncytialization, and EVTs through a process called EMT. During the formation of the STs, some of the CTs proliferate and then fuse. The other CTs that transition into the EVTs first proliferate and then differentiate to form PCTs (highly proliferative and non-invasive cells), which in turn differentiate into DCTs (non-proliferative and highly invasive cells). The DCTs then break through the overlying ST layer and anchor the embryo to the uterus. From the anchoring villi, the DCTs differentiate into iEVTs which further invade the decidua and the inner one-third of the myometrium. In the decidua, some of the iEVTs invade the glands and the spiral arteries. Those that invade the glands are called egEVTs while those that invade the spiral arteries are called evEVTs. The invasion and transformation of the maternal spiral arteries into low resistant vessels by the evEVTs enable a steady, low-pressure flow of blood into the intervillous space, establishing the placenta as the primary system for feto-maternal exchanges. When the remaining iEVTs reach the myometrium, they cluster and merge to form multinucleated giant cells, thereby losing their invasive capacity to prevent over-invasion. Abbreviations: CTs, cytotrophoblasts; PCTs, proximal column trophoblasts; DCTs, distal column trophoblasts; iEVTs, interstitial extravillous trophoblasts; egEVTs, endoglandular extravillous trophoblasts; evEVTs, endovascular extravillous trophoblasts

Human placentation is a complex and dynamic developmental process that begins upon the apposition and adhesion of the blastocyst to the uterine lining, thus establishing the intricate feto-maternal interface [1]. Following adhesion, the specialized outer cells of the blastocyst, known as the trophectoderm, differentiate into mononuclear, actively proliferating cells called cytotrophoblasts (CTs). These CTs function as the primary stem cells for the subsequent development of the other trophoblast lineages via two main pathways: the villous pathway and the extravillous pathway [2]. Along the villous pathway, the CTs multiply, push outward and fuse with one another to form an outer, multinucleated lineage called the syncytiotrophoblast (ST), which serves as the barrier that controls physiological exchanges between the mother and the fetus [3, 4]. (Fig. 1).

The CTs continue to proliferate to form primary chorionic villi, which are subsequently invaded by extraembryonic mesoderm to form secondary villi, and later develop fetal capillaries to become tertiary villi. At the tips of these tertiary villi where they contact the maternal uterine tissue, the CTs break through the ST layer and form a continuous layer called the CT shell. This anchors the developing placenta to the maternal decidua, thus commencing differentiation along the extravillous pathway. From these anchoring villi, the CTs differentiate into a highly invasive lineage known as the extravillous trophoblasts (EVTs). The proliferation of the CTs first generate intermediary cells called proximal column trophoblasts (PCTs). These mitotically-active cells remain closer to the main body of the villous tree [4]. However, some of them migrate distally and differentiate to form non-mitotic cells called distal column trophoblasts (DCTs) which are the precursor of the EVTs. The DCTs migrate and then enter the decidua basalis where they differentiate into interstitial EVTs (iEVTs) [4](Fig. 1).

As they navigate through the dense maternal uterine tissue, the iEVTs encounter various maternal structures and initiate profound physiological changes essential for a successful pregnancy. Some of the iEVTs differentiate into specialized cells called endoglandular EVTs (egEVTs) which invade and modify the uterine glands. By opening these glands directly into the developing intervillous space, the egEVTs ensure a continuous supply of nutrient-rich glandular secretions, sustaining the embryo before the full onset of maternal blood circulation [4]**(**Fig. 1). As this is taking place, some iEVTs form dense cellular plugs within the lumens of the spiral arteries. These plugs act as temporary physical barriers that restrict maternal blood-flow into the placenta [5], which helps maintain a low-oxygen environment within the intervillous space. This hypoxic state is not detrimental; rather, it is a required condition that facilitates and directs the necessary early phases of trophoblast development and ongoing placentation [6] without causing oxidative stress damage to the delicate early villous tissues.

As placentation progresses, the critical physiological event known as spiral artery remodeling is initiated to accommodate the exponentially increasing metabolic demands of the growing fetus. This transformation occurs in two overlapping phases largely mediated by the iEVTs.

In the first phase, the iEVTs invade the spiral arteries in the decidua and myometrium as a new form of EVTs called endovascular EVTs (evEVTs). These cells begin to break down the surrounding extracellular matrix, degrade the musculoelastic tissue, and induce apoptosis in the vascular smooth muscle cells of the spiral arteries. As they modify the structural integrity of the vessel walls, these evEVTs secrete a specialized, amorphous fibrinoid material containing fibronectin and laminin, effectively embedding themselves within the modified arterial wall and paving the way for the second phase of remodeling [4].

The second phase is characterized by the retrograde migration of the evEVTs. These cells migrate backward up the lumen of the spiral arteries against the direction of blood-flow, directly replacing the native maternal endothelial cells lining the vessels. As a direct result of this dual interstitial and endovascular invasion and modification, the spiral arteries lose their natural contractility and thick muscular walls. They transform from narrow, high-resistance, coiled vessels into wide, flaccid, and tortuous low-resistance conduits [7, 8]. This critical physiological adaptation ensures that large volumes of maternal blood can flow smoothly and safely into the intervillous space at a lower perfusion pressure, delivering optimal oxygen and nutrients directly to the developing fetus while protecting the delicate villi from structural damage. This process is called uteroplacental circulation, and is fully established by the 12th week of gestation. At this stage, the embryo is called a fetus.

## Overview of E-cadherin

**Fig. 2:**
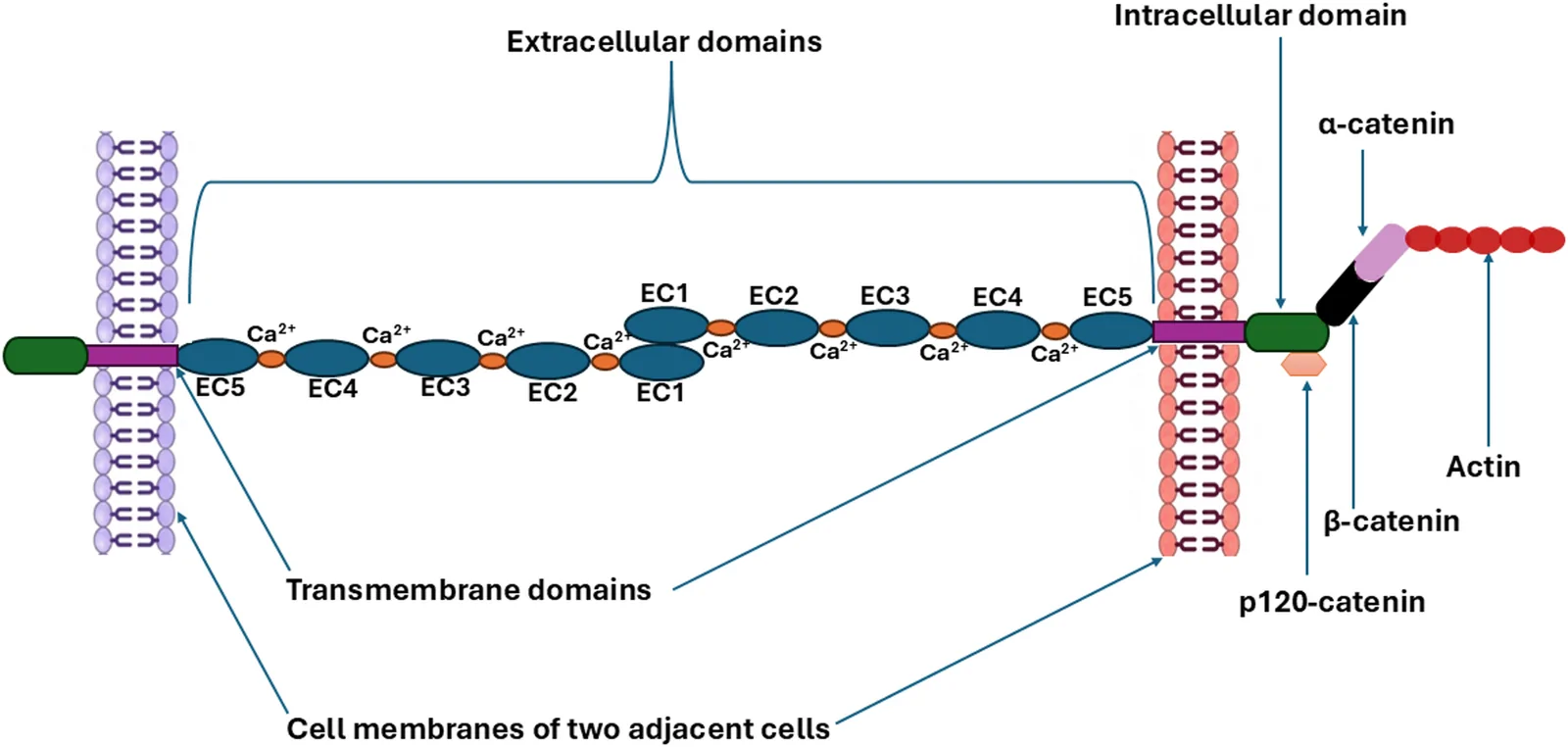
Structural organization and cellular role of the E-cadherin adhesion complex. This diagram illustrates E-cadherin, a critical transmembrane glycoprotein that drives calcium-dependent cell-to-cell adhesion in epithelial tissues. The extracellular region consists of five tandemly repeated domains (EC1–EC5) that form homophilic, zipper-like bonds with other E-cadherin molecules protruding from an adjacent cell. This intercellular bridging relies heavily on calcium ions (Ca²⁺), which bind between successive EC units to keep the extracellular projection rigid and functional. Structurally, the protein passes through the plasma membrane via a single transmembrane domain, extending into a cytoplasmic tail inside the cell. This intracellular portion binds to a network of catenin proteins — p120-catenin, β-catenin, and α-catenin — which links the adhesion complex to the internal actin cytoskeleton. This dual connection preserves tissue architecture and maintains cell polarity under normal conditions, whereas its disruption or loss triggers EMT, leading to cell dissociation and increased cellular motility. Abbreviations: EC, extracellular cadherin; EMT, epithelial-mesenchymal transition

E-cadherin is a 120 kDa transmembrane glycoprotein that is encoded by the *CDH1* gene. It serves as a fundamental determinant of epithelial architecture and tissue stability. Structurally, the mature protein spans the plasma membrane via a single transmembrane segment, and bridges the extracellular environment with the intracellular environment (Fig. 2).

E-cadherin consists of three domains: extracellular domain (ectodomain), transmembrane domain and intracellular domain (cytoplasmic domain). The extracellular domain is large and is characterized by five tandemly repeated subunits that require calcium ions to achieve the structural rigidity needed for homophilic binding. Through these specific and bridge-like interactions, E-cadherin molecules on adjacent cells bind directly to one another, thus functioning as part of the adhesion molecules that seal the spaces between cells. The intracellular domain is a highly conserved cytoplasmic tail which serves as a structural and signaling epicenter for epithelial cells. It connects to the actin component of the cytoskeleton by binding to adaptor proteins: p120-catenin, β-catenin, and α-catenin. The transmembrane domain is a single-pass, α-helical segment consisting of approximately 23 to 25 hydrophobic amino acids. It anchors the protein within the lipid bilayer of the cell membrane and so connects the extracellular domain to the intracellular domain. This core cadherin-catenin complex undergoes continuous, highly regulated turnover on the cell surface, providing the structural plasticity required for dynamic cellular remodeling while maintaining the overall integrity of the epithelial barrier (Fig. 2).

Beyond its role as an adhesion molecule, E-cadherin is an essential orchestrator of cellular organization and polarity [9]. By tightly regulating how cells arrange themselves relative to one another, it drives the development of apico-basal polarity, ensuring that the apical surface of an epithelial sheet remains structurally and functionally distinct from its basal counterpart. This precise spatial orientation is vital for the physiological functions of complex barrier organs, including the skin, kidneys, and intestines, where the epithelium must simultaneously block the entry of pathogens and toxins while preventing the uncontrolled leakage of internal fluids.

In contemporary cell biology, E-cadherin is no longer considered as a mere passive structural anchor, but rather as a sophisticated, bidirectional molecular signaling hub that coordinates complex tissue behaviors from embryonic development through adulthood [10]. It serves as a critical signaling epicenter for contact inhibition, a physiological process where physical contact between proliferating epithelial cells transmits inhibitory cues that halt further cell division. This regulatory mechanism is tightly coupled with the spatial sequestration of beta-catenin [11]. When cell-cell adherens junctions are stable, beta-catenin remains bound at the plasma membrane and hence prevented from entering the interior of the cell. However, when E-cadherin expression drops or junctions degrade, the freed pool of beta-catenin translocates to the cell nucleus [12], where it binds to transcription factors and activates growth-promoting genes that drive proliferation.

Due to this dual capacity to restrict mechanical detachment and suppress mitogenic signaling, E-cadherin is widely classified as a potent tumor suppressor [13]. During the progression of numerous human carcinomas, neoplastic cells frequently circumvent these regulatory constraints by initiating a process known as the epithelial-to-mesenchymal transition (EMT) [14], which is a pathological process in the context of carcinogenesis. During EMT, transcriptional reprogramming actively shuts down E-cadherin expression, dismantling the cadherin-catenin complex. The loss of this critical brake on cellular motility allows malignant cells to shed their epithelial characteristics, detach from the primary tumor mass, and acquire a mesenchymal phenotype [15]. Currently, understanding the complex molecular pathways that govern E-cadherin expression and turnover remains a central focus in identifying therapeutic targets to disrupt cancer metastasis and tissue fibrosis. Perhaps, in the future, E-cadherin will receive a similar attention as a therapeutic target in placental pathology.

## E-cadherin and trophoblast differentiation along the villous pathway

As trophoblasts differentiate along the villous pathway, in a process called trophoblast fusion or syncytialization, the mononucleated CTs combine by dissolving their adjacent cell membranes and unifying their cytoplasm. These individual cells integrate into a single, continuous, multinucleated layer known as the ST [4, 16, 17](Fig. 1). Poorly formed ST layer is implicated in the pathogenesis of preeclampsia [18] and fetal growth restriction [19, 20].

An investigation conducted by Dr. C. Coutifaris and his research team was the first to report that E-cadherin is a critical transmembrane protein responsible for initiating the clustering of CTs [21]. By examining the laboratory-controlled aggregation of human primary CTs, the investigators observed that E-cadherin was localized on the cell surface at points of cell-cell contact. There was a time-dependent increase in E-cadherin levels, which coincided with maximal aggregate formation of the cells at 24 h. However, this was followed by a significant reduction of E-cadherin expression when cellular fusion was completed [21]. Furthermore, treatment of BeWo cells with 8-bromo cyclic AMP (cAMP) induced cellular fusion and syncytium formation, and this was accompanied by the disappearance of E-cadherin from the cell-cell surface. The researchers eventually concluded that E-cadherin exists in a dynamic state in fusion-competent CTs and that its downregulation coincides with trophoblast fusion in a potentially cAMP-dependent manner [21]. In a follow-up study, it was demonstrated that the loss of E-cadherin expression is a requirement for human trophoblast fusion, showing that while high levels of E-cadherin are necessary to physically aggregate mononucleated CTs, this protein must be dynamically downregulated to allow cell membranes to disappear for cytoplasmic merging to occur [22].

During forskolin-induced BeWo cell fusion, E-cadherin was found to be downregulated by Twist, which is a recognized transcription factor that localizes to the ST nuclei [23]. Furthermore, another transcription factor called Snail, which is a known repressor of E-cadherin, was observed to have an inverse correlation with E-cadherin during BeWo cell fusion [24]. While the ability of Snail to repress E-cadherin during trophoblast fusion remains to be confirmed, the findings suggest that Twist is a transcriptional repressor of E-cadherin during trophoblast fusion.

Following these findings, it remained unknown whether the dynamic mechanical forces that arise during villous development affect fusion. This knowledge gap was subsequently bridged when it was later demonstrated that mechanical stress patterns arise around sites of localized fusion in cell monolayers and in patterns that match computational predictions of villous morphogenesis. When these mechanical stress patterns were applied to CT monolayers, it became apparent that equibiaxial compressive stresses facilitate the downregulation of E-cadherin during trophoblast fusion [25]. Since biophysical models suggest that equibiaxial compressive stress lowers the energetic barrier for membrane fusion by driving lipid and protein rearrangement, these mechanical perturbations may lower the energetic barrier for membrane coalescence by facilitating the spatial rearrangement of lipids and proteins at the fusion interface. However, this biophysical mechanism remains largely theoretical in the context of live trophoblasts. Future studies should utilize advanced imaging techniques, such as Fluorescence Lifetime Imaging Microscopy (FLIM) or Laurdan dye staining, to measure real-time membrane fluidity and lipid raft dynamics under applied mechanical stress. Coupling these with atomic force microscopy (AFM) will help quantify the exact mechanical tension changes at the cell-cell interface during E-cadherin disappearance.

**Fig. 3:**
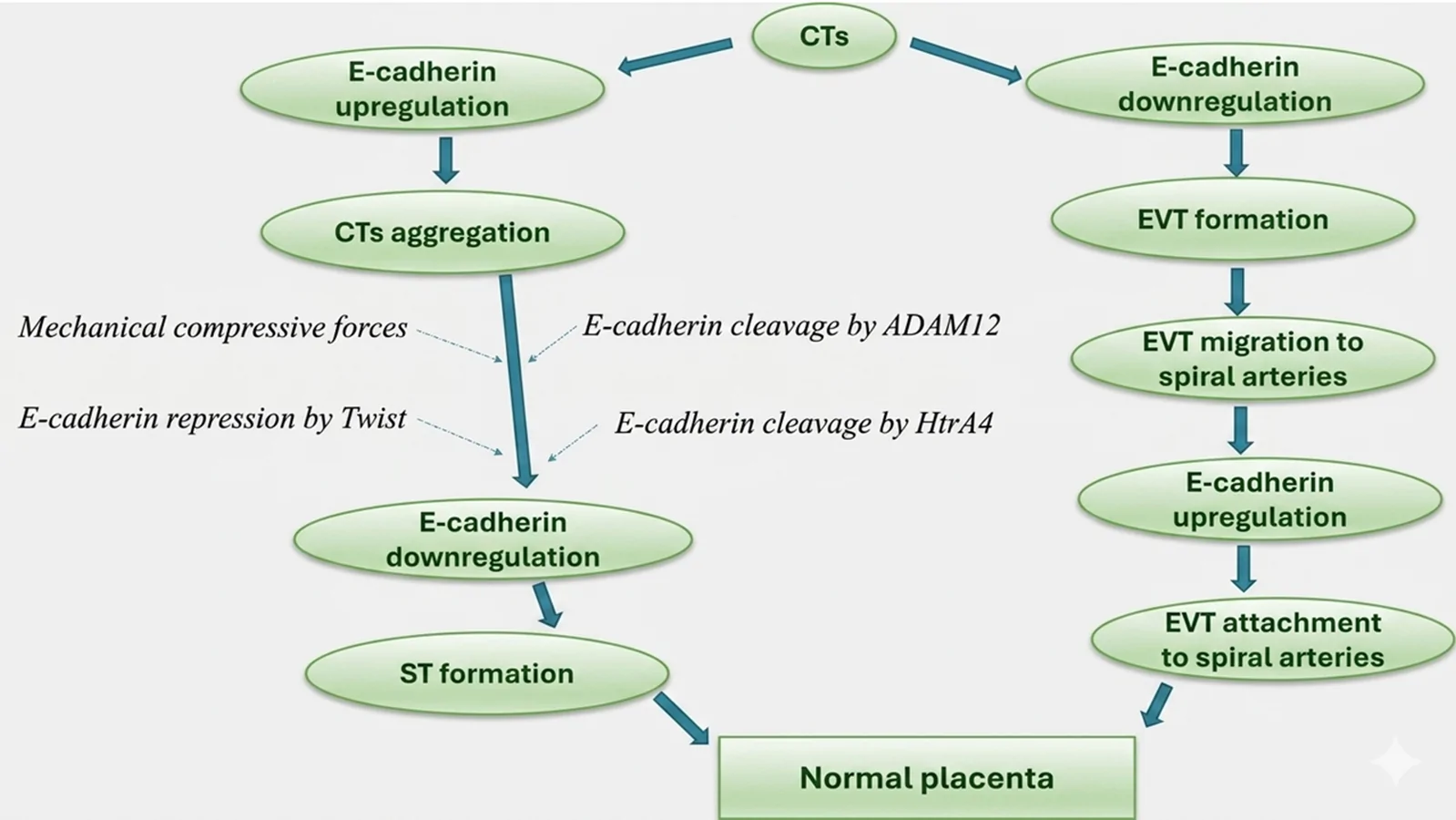
Illustration of the distinct regulation of E-cadherin expression pattern during human placentation. The diagram serves as a conceptual and hypothetical roadmap showing where various upstream inputs are proposed to act on the E-cadherin timeline during lineage transitions. During trophoblast fusion, there is a transient upregulation of E-cadherin to aggregate the CTs. Then, E-cadherin is downregulated via mechanical compressive forces, transcriptional repression by Twist and through enzymatic cleavage by ADAM12 and HtrA4 to induce fusion. On the other hand, during the formation of the EVTs, E-cadherin expression in the CTs is reduced to enhance migration, and when the EVTs arrive at the location of the spiral arteries, E-cadherin expression increases to enhance the attachment of the iEVTs to the spiral arteries for remodeling. Abbreviations: ADAM12, A Disintegrin And Metalloprotease metallopeptidase domain 12.; HtrA4, High-temperature requirement factor A4; CTs, cytotrophoblasts; EVTs, extravillous trophoblasts; iEVTs, interstitial extravillous trophoblasts

Beyond the transcriptional and mechanical influences, investigations into the regulatory pathways governing this trophoblast transition stage have identified HtrA4 as a critical mediator that influences the downregulation of E-cadherin. When HtrA4, which is an enzyme highly expressed in the placenta, was silenced or deleted in a fusion model, the CTs failed to downregulate E-cadherin [26, 27]. In these knockout models, E-cadherin expression persisted thus completely blocking cell-to-cell fusion and the formation of a syncytium. This indicates that HtrA4 facilitates the downregulation of E-cadherin during trophoblast fusion. Moreover, trophoblast fusion has been reported to be actively potentiated by ADAM12-mediated ectodomain shedding of E-cadherin, which serves as a molecular switch to destabilize existing adherens junctions and catalyze the transition toward membrane coalescence [28]. Beside this, the dramatic redistribution and subsequent reduction of E-cadherin has been observed to facilitate a reorganization of the actin cytoskeleton [29]. This enzymatic cleavage of the extracellular domain and the remodeling of the cortical cytoskeleton likely provide a localized mechanism to alleviate mechanical constraints at the cell-cell interface, thereby enabling the subsequent expression and activation of syncytins to finalize the syncytialization process. This potential mechanism requires formal experimental validation. To advance our understanding of this E-cadherin pathway beyond current baseline paradigms, researchers must address several critical mechanistic gaps. While ADAM12 effectively cleaves the extracellular domain of E-cadherin, the precise intracellular trafficking pathways — specifically the involvement of clathrin-mediated endocytosis versus proteasomal or lysosomal degradation machinery — responsible for clearing the remaining cytoplasmic protein pool remain to be elucidated. With current literature identifying HtrA4, ADAM12, and compressive forces as independent upstream regulators of E-cadherin downregulation (Fig. 3), it remains to be determined if these pathways operate in a linear cascade or parallel networks. Researchers should investigate if mechanical strain directly triggers the expression or enzymatic activation of HtrA4 and ADAM12. Identifying whether mechanosensitive ion channels such as PIEZO1 sense these compressive fields and translate them into the intracellular cAMP signals that initiate the fusion cascade will bridge a major signaling gap.

Taken together, the research findings indicate that a high expression of E-cadherin facilitates the aggregation of the CTs while its downregulation is a precedent to the fusion of the cells, rather than being a terminal outcome. When this cell adhesion protein abnormally persists on the ST layer, it signifies incomplete fusion. Hence, in histological analysis, the disappearance of E-cadherin-stained cell boundaries is a reliable indicator of successful syncytial maturation while quantifying the levels of E-cadherin serves as a valid experimental metric to evaluate trophoblast fusion efficacy. However, quantifying the levels of E-cadherin should be considered a complementary molecular metric rather than a standalone measure of fusion efficiency. Persistent apical expression of E-cadherin is correlated with preeclampsia and fetal growth restriction but its utility as a predictive biomarker or therapeutic target remains untapped. Large-scale translational studies should determine whether the levels of soluble E-cadherin fragments shed by ADAM12 are detectable in maternal blood during the first trimester.

## E-cadherin and trophoblast differentiation along the extravillous pathway

Trophoblast differentiation along the extravillous pathway occurs as the CTs differentiate into PCTs. These PCTs then differentiate into DCTs, which further differentiate into iEVTs. Later, the iEVTs differentiate into egEVTs and evEVTs (Fig. 1). This EVT lineage transition, described as physiological EMT, represents a specialized developmental program that is strictly regulated by homeostatic uterine cues [30], distinguishing it from unconstrained pathological EMT cascades. This process is characterized by the dynamic expression of adhesion molecules, especially the downregulation of E-cadherin during the transition toward an invasive phenotype [4]. The transient reduction in E-cadherin expression destabilizes epithelial cell-cell junctions, effectively lowering the adhesive barriers that otherwise constrain trophoblast movement. This molecular remodeling facilitates the transition of trophoblast cells from a cohesive epithelial state to a migratory phenotype [4]. Therefore, it is a hallmark of the physiological EMT required for invasive trophoblasts to detach from anchoring cell columns and invade the decidua.

Although E-cadherin is detected across CTs, PCTs, DCTs, iEVTs, egEVTs and evEVTs, it is expressed in varying levels [4]. These variable expression levels are among the key determinants of cell motility: High E-cadherin expression means stationary, tightly bound barrier cells, whereas low E-cadherin expression means highly mobile, invasive cells. The requirement of this cell-surface down-regulation to unleash trophoblast motility was demonstrated in implantation-site derived intermediate trophoblast models [31]. When E-cadherin expression was experimentally restored in invasive trophoblasts, the cells lost their individual migratory shapes, reverted to cohesive cellular aggregates, and exhibited a robust, contact-dependent inhibition of cellular motility [31]. This functional evidence suggest that the down-regulation of E-cadherin is a likely requirement for EVT migration into the decidua.

The downregulation of E-cadherin in invasive cells is mediated by repressors such as Twist, Snail, ZEB1 and ZEB2. Therefore, the differentiation of the CTs into the EVTs is likely to be similarly mediated by these repressors. However, in EVTs, the available evidence only demonstrate an inverse relationship between E-cadherin and these repressors [32, 33], leaving a knowledge deficit that warrants further mechanistic study. Nevertheless, this distinct cellular plasticity is supported by the specific upregulation of N-cadherin, which actively promotes the disassembly of E-cadherin-mediated cellular adhesions to drive the invasive capacity of the trophoblasts. Dysregulation of this molecular switch, characterized by the persistent expression of E-cadherin or the failure to initiate mesenchymal transformation, is strongly associated with pathologies such as preeclampsia and fetal growth restriction [4, 34, 35].

As the EVTs invade the decidua, they undergo a specialized “vascular mimicry”. During this process, E-cadherin is replaced by endothelial-specific adhesion molecules, including VE-cadherin and VCAM-1, enabling the remodeling of maternal spiral arteries [34]. This adaptive switch to an endothelial-like adhesion phenotype is fundamental to successful feto-maternal cellular integration within the uterine vasculature [35, 36]. It was reported that EVT lineages closer to the placental-myometrial interface have less E-cadherin expression than those found deeper in the myometrium [36]. This indicates that actively migrating iEVTs at the myometrial surface express lower levels of E-cadherin to facilitate invasion, while deeper, settled myometrial evEVTs exhibit higher, restored E-cadherin expression. This re-expression likely reflects the functional transition from a migratory state to a stabilized vascular phenotype, as these cells replace the maternal endothelial lining. Within this framework, E-cadherin cooperates with VE-cadherin and VCAM-1 to mediate homotypic cell-to-cell attachment and aggregation among the settled endovascular population, relying on VE-cadherin to mediate trophoblast-endothelial cell interactions and displacement while VCAM-1 interacts with α4 integrins to support adhesion to the vascular microenvironment and withstand the hemodynamic forces of maternal blood flow [35, 37, 38]. Such partial reversal of EMT suggests that the loss of E-cadherin is not merely a terminal commitment but a transient state required to facilitate the replacement of resident maternal endothelium. Ultimately, this spatio-temporal expression pattern of E-cadherin serves as a key determinant ensuring that EMT remains tightly restricted to the physiological requirements of the uterine environment.

Regardless of these findings, a lot remains to be studied. It is unknown how epigenetic or mechanical triggers signal actively invading cells to suddenly stop, change phenotype and switch on E-cadherin once they invade the maternal vessels. Investigations of how the microenvironmental and mechanical cues such as changes in shear stress from maternal blood-flow or localized oxygen gradients act as the “molecular brakes” to regulate this process is highly recommended.

While the transcriptional downregulation of E-cadherin is widely recognized as a hallmark of the EMT phenotypic switch, the precise biochemical mechanisms governing the post-translational proteolytic processing of E-cadherin during CT differentiation into EVTs remain an uncharted frontier. In oncology and neurobiology, it is known that E-cadherin is subject to sequential regulated intramembrane proteolysis (RIP) that is initiated by disintegrin and metalloproteinases (primarily ADAM10 and ADAM12) and completed by the gamma-secretase (presenilin-1) complex. However, whether this precise proteolytic machinery operates in invading trophoblasts, and how it is spatio-temporally regulated, is yet to be reported. Future studies must systematically profile the expression, localization, and activation kinetics of candidate sheddases within the decidua. Utilizing high-resolution spatial transcriptomics and multiplexed co-immunofluorescence on first-trimester placental tissue will be essential to map the physical microenvironments where these proteases colocalize with full-length E-cadherin. In cancer biology, it is largely known that proteolytic cleavage of E-cadherin does not only remove a physical tether; it generates two highly bioactive fragments which directly promote cell migration. For instance, soluble E-cadherin acts as a paracrine/autocrine signal that can bind and activate EGFR or HER2 pathways to accelerate migration. However, regarding EVT functions, the roles of these fragments are unknown. Future research should find out if trophoblast-derived soluble E-cadherin functions in a similar signaling capacity, or if it acts as a competitive decoy to disrupt maternal epithelial cell-cell junctions in the decidua.

In reality, the placental bed is highly heterogeneous. Recent advancements in spatial transcriptomics show that trophoblasts do not migrate as a uniform wave; they exist in distinct micro-niches. Hence it is suggested that further studies will utilize single-cell RNA sequencing (scRNA-seq) combined with multiplex spatial proteomics to map the precise, cell-by-cell co-expression of E-cadherin alongside localized immune cells (like uterine Natural Killer cells). This will reveal how the local immune microenvironment shapes the cadherin switch.

Extracellular matrix stiffness changes dramatically from the decidua to the deeper myometrium. E-cadherin is not just an adhesion molecule; it is also a mechanosensor linked to the actin cytoskeleton. Investigating how physical tissue density and ECM stiffness modulate E-cadherin expression will bridge the gap between biomechanics and placental pathology.

## Conclusions

E-cadherin serves as a pivotal and microenvironmentally sensitive molecular switch that characterizes both the villous and extravillous trophoblast differentiation pathways during human placentation. Its precise downregulation is required for both syncytialization and physiological EMT (Table 1). Conversely, its subsequent upregulation during deep invasion reflects a partial reversal of EMT, facilitating stable integration and remodeling of the maternal spiral arteries (Fig. 3). Dysregulation of this expression pattern potentially restricts vital lineage transitions, contributing to defective syncytialization and the shallow invasion characteristic of preeclampsia and fetal growth restriction. Despite the multiple lines of evidence regarding the expression pattern of E-cadherin during trophoblast differentiation, there is a dearth of knowledge about the underlying mechanisms. In the future, transitioning from descriptive profiling to functional, multi-disciplinary mechanisms — specifically leveraging spatial transcriptomics, advanced biophysical modeling and mechanosensitive channel tracking — will be essential to mapping the mechanistic roles of E-cadherin in placental development and disease.

**Table 1:** Critical Synthesis of Experimental Evidence Supporting E-cadherin expression dynamics during trophoblast differentiation

Reference	Experimental Model System	Gestational Stage at which the placenta was obtained	Experimental Manipulation / Stimulus	Observed E-cadherin Dynamics	Biological Outcome / Lineage Transition	Tier & Strength of Evidence
Coutifaris et al. 1991	Primary human villous CTs & BeWo choriocarcinoma cell line	Third trimester	Spontaneous in vitro aggregation/fusion; 8-bromo cyclic AMP (cAMP) treatment	Time-dependent increase during aggregation followed by reduction upon fusion completion	Direct expression pattern as cells aggregated but inverse expression as cells fused	Associative
Getsios et al. 2000	Primary human villous CTs	Third trimester	Spontaneous in vitro aggregation and cellular syncytialization	Downregulation correlates with β-catenin release and subsequent nuclear translocation	Inverse expression pattern during fusion	Associative
Ng et al. 2011	BeWo cell line	N/A	Forskolin-induced cellular fusion; Twist siRNA and exogenous expression vectors	Downregulated via transcriptional repression directed by Twist; inverse correlative profile tracked with Snail	Inverse expression pattern showing that Twist reduces E-cadherin levels during fusion	Associative
Parameshwar et al. 2024	Cytotrophoblast cell monolayers (Biomechanical model)	N/A	Application of equibiaxial compressive stress fields	Facilitated spatial rearrangement and accelerated disappearance from cell junctions	Expression reduction demonstrating mechanical compressive strain triggers E-cadherin loss during fusion	Associative
Mansilla et al. 2021	Trophoblast cell fusion model	First & third trimester	Silencing or genetic deletion (CRISPR/Cas9 knockout) of HtrA4 enzyme	Persistent surface expression; failure to downregulate E-cadherin	Expression reduction showing that HtrA4 is required to clear E-cadherin during fusion	Associative
Aghababaei et al. 2015	Trophoblast cell fusion model	First Trimester	ADAM12 siRNA-directed knockdown and over-expression vectors	Potentiated ectodomain shedding and dramatic surface protein reduction	Expression reduction demonstrating ADAM12 cleaves the extracellular domain of E-cadherin during fusion	Associative
Shih et al. 2002	Implantation site-derived intermediate trophoblast cell line (IST-1)	First Trimester	Adenoviral-mediated expression vector restoration (E-cad/Ad5); function-perturbing antibodies	Artificial cell-surface re-expression of E-cadherin on invasive phenotypes	Functional role proving E-cadherin directly suppresses cell motility via contact-dependent inhibition	Functional
Floridon et al. 2000	Placental tissue biopsies (Intrauterine, ectopic, molar)	First and third trimester	Spatial co-immunofluorescence mapping / tissue profiling	Variable spatial expression across distinct CT, column, and EVT sub-populations	Spatial E-cadherin expression pattern in trophoblast subtypes	Associative
Duzyj et al. 2015	Placental bed tissue biopsies (Placenta accreta vs. normal)	Third trimester	Ex vivo histopathological tissue profiling	Loss of E-cadherin expression in deep, highly invasive myometrial EVT sub-populations	Spatial E-cadherin expression pattern among EVT subtypes	Associative

Legend: Studies are organized into distinct tiers based on the target of experimental manipulation to separate upstream regulatory networks from validation of the adhesion complex. Associative Evidence designates investigations where there was either descriptive tissue mapping, or the manipulation of upstream transcriptional factors (*Twist*), enzymatic machinery (*HtrA4*, *ADAM12*), or biophysical parameters (*Mechanical compressive forces*). While genetic or biomechanical models involve active interventions, their findings remain associative regarding E-cadherin’s autonomous properties because manipulation of these upstream targets potentially triggers pleiotropic downstream actions independent of E-cadherin, meaning the observed biological outcomes cannot be isolated to E-cadherin alterations alone. Conversely, Functional Evidence is strictly reserved for investigations where the E-cadherin protein or *CDH1* gene structure itself was targeted (via expression vectors, rescue assays, or function-perturbing antibodies) to isolate and demonstrate its explicit, independent role in a biological process, such as contact-dependent inhibition of cell motility

Abbreviations: ADAM12, A Disintegrin And Metalloprotease 12; cAMP, 8-bromo cyclic adenosine monophosphate; CTs, cytotrophoblasts; EVT, extravillous trophoblast; EMT, epithelial-mesenchymal transition; HtrA4, High-temperature requirement factor A4; N/A, not applicable

## Data Availability

No datasets were generated or analysed during the current study.
